# Cystathionine γ-lyase is expressed in human atherosclerotic plaque microvessels and is involved in micro-angiogenesis

**DOI:** 10.1038/srep34608

**Published:** 2016-10-06

**Authors:** J. C. van den Born, R. Mencke, S. Conroy, C. J. Zeebregts, H. van Goor, J. L. Hillebrands

**Affiliations:** 1 Department of Pathology and Medical Biology - Division of Pathology, University of Groningen, University Medical Center Groningen. Hanzeplein 1 9700 RB Groningen, the Netherlands; 2 Department of Surgery - Division of Vascular Surgery, University of Groningen, University Medical Center Groningen. Hanzeplein 1 9700 RB Groningen, the Netherlands

## Abstract

Atherosclerotic plaques are classically divided into stable and vulnerable plaques. Vulnerable plaques are prone to rupture with a risk for infarction. High intraplaque microvessel density predisposes to plaque vulnerability. Hydrogen sulfide (H_2_S) is a proangiogenic gasotransmitter which is endogenously produced by cystathionine γ-lyase (CSE), and is believed to have vasculoprotective effects. However, due to its proangiogenic effects, H_2_S may result in pathological angiogenesis in atherosclerotic plaques, thereby increasing plaque vulnerability. The aim of this study was to determine CSE expression pattern in atherosclerotic plaques, and investigate whether CSE is involved in micro-angiogenesis *in vitro*. Endarterectomy plaques were studied for CSE expression, and the role of CSE in micro-angiogenesis was studied *in vitro*. CSE is expressed in plaques with similar levels in both stable and vulnerable plaques. CSE co-localized with von Willebrand Factor-positive microvessel endothelial cells and alpha-smooth-muscle actin-positive SMCs. *In vitro*, inhibition of CSE in HMEC-1 reduced tube formation, cell viability/proliferation, and migration which was restored after culture in the presence of H_2_S donor GYY4137. CSE is expressed in intraplaque microvessels, and H_2_S is a stimulator of micro-angiogenesis *in vitro*. Due to this pro-angiogenic effect, high levels of CSE in atherosclerotic plaques may be a potential risk for plaque vulnerability.

Cardiovascular diseases are the predominant cause of death in developed countries^1^. The main cause is atherosclerosis, a chronic vascular disease characterized by plaque formation. Depending on the cellular and matrix composition, plaques can be classified as stable or unstable^2^. Stable plaques cause luminal narrowing but are less prone to rupture, whereas unstable/vulnerable plaques are prone to rupture, resulting in ischemic stroke and myocardial infarction. Intraplaque angiogenesis (*i.e.*, high microvessel density) is associated with intraplaque hemorrhage and plaque vulnerability, resulting in symptomatology^3^,^4^.

Angiogenesis is stimulated by hydrogen sulfide (H_2_S), a gaseous signaling molecule^5^,^6^. H_2_S is recognized as the third gasotransmitter next to nitric oxide (NO) and carbon monoxide (CO)^7^. H_2_S is endogenously produced by three enzymes cystathionine γ-lyase (CSE), cystathionine β-synthase (CBS), and 3-mercaptopyruvate sulfurtransferase (3MST). In vascular tissue, CSE is considered the major H_2_S synthesizing enzyme. H_2_S induces vasodilatation, reduces inflammation, inhibits platelet aggregation and scavenges reactive oxygen species^8^,^9^,^10^,^11^.

The proangiogenic effects of H_2_S have been demonstrated in experimental model systems *in vivo* as well as *ex vivo*. Administration of the CSE inhibitor D,L-propargylglycine (PPG) resulted in inhibition of angiogenesis in chorioallantoic membrane^6^. Moreover, VEGF-induced angiogenesis was absent in aortic ring explants from CSE-deficient mice indicating that angiogenesis is CSE-dependent in this model^12^.

Systemic administration of H_2_S donors is vasculoprotective in experimental models for cardiovascular disease^13^. Administration of sodium hydrosulfide (NaHS) reduced atherosclerotic plaque size in ApoE^−/−^ mice^14^. Furthermore, CSE deficiency aggravated atherosclerotic plaque formation and smooth muscle cell proliferation, an effect which could be rescued by administration of NaHS^15^,^16^,^17^.

In the early phase of plaque development (*i.e.* endothelial dysfunction and fatty streak formation) the CSE/H_2_S pathway is clearly vasculoprotective. However, during plaque progression, composition of the plaque becomes increasingly important in terms of vulnerability. Since H_2_S is a known pro-angiogenic molecule, we hypothesize that local intraplaque H_2_S production could aggravate plaque vulnerability by promoting intraplaque angiogenesis. To our best knowledge, no data exist about endogenous H_2_S production in human macrovasculature and atherosclerosis.

The aim of the present study is to determine CSE expression in atherosclerotic plaques, and the role of CSE-derived H_2_S in micro-angiogenesis. To investigate this, we studied human carotid endarterectomy plaques for CSE protein expression. Furthermore, the effect of inhibition of CSE expression and activity, and subsequent rescue by exogenous H_2_S, on HMEC-1 micro-angiogenesis, proliferation and migration was studied *in vitro*.

## Results

### Patient characteristics

Patient characteristics (n = 19) at time of carotid endarterectomy (CEA) are shown in Table 1. Most of the patients were male (79%). The mean age of all patients was 72.2 ± 8.1 yrs. According to the guidelines, 15 patients were overweight (BMI > 25 kg/m^2^), and 8 patients suffered from obesity (BMI > 30 kg/m^2^), with a mean BMI of 28.7 ± 3.5. Systolic blood pressure was 146 ± 14 mmHg, and diastolic blood pressure was 72 ± 12 mmHg.

### Atherosclerotic plaque characteristics

**Figure 1:**
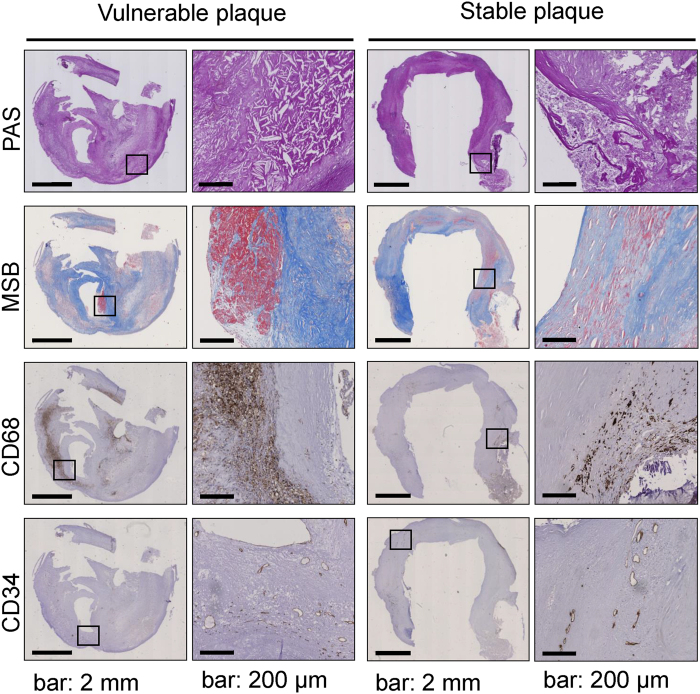
Plaque morphology. Representative photomicrographs of a vulnerable plaque (**A**,**C**,**E**,**G**) and a stable plaque (**B**,**D**,**F**,**H**). Images show narrowed lumen (PAS staining) (**A**,**B**) and cholesterol clefts. Fibrous cap and connective tissue was visualized with MSB staining (blue) (**C**,**D**). Fibrin, indicating an intraplaque hemorrhage, is stained red. Inflammatory cells, identified by positive CD68 staining are shown in panels (**E**,**F**). Intraplaque microvessels were characterized by positive CD34 staining (**G**,**H**). Scalebar 2 mm in overview photomicrographs and 200 μm in detailed pictures.

The carotid plaque specimens were divided into either vulnerable or stable based on the criteria described in the *Methods* section. Criteria included the following parameters: fibrous cap thickness, CD68^+^ macrophage infiltration, density of intraplaque microvessels, and the presence of intraplaque hemorrhage. Figure 1 displays representative examples of a vulnerable and stable plaque. Vulnerable plaques were characterized by cholesterol cleft abundance, intraplaque hemorrhage, macrophage infiltration and intraplaque angiogenesis. To validate our plaque classification, plaques depicted in Fig. 1 were also classified according to the Oxford plaque classification^18^. According to this classification the overall stability of the vulnerable plaque would be “unstable with ruptured cap” due to the presence of a thrombus. The fibrous cap in the stable plaque is >200 μm at the thinnest and therefore scored “stable” according the Oxford Plaque Classification^19^. Our classification and the Oxford Plaque Classification thus has similar outcome with respect to plaque stability. Out of 19 CEA plaques, 8 were classified as vulnerable. No significant differences were observed in patient characteristics between patients with stable and vulnerable plaques (Table 1). However, 3 out of 4 plaques from asymptomatic patients were classified as stable, suggesting that in our patient cohort asymptomatology is associated with plaque stability.

### CSE is expressed in intraplaque microvessel endothelial cells

**Figure 2:**
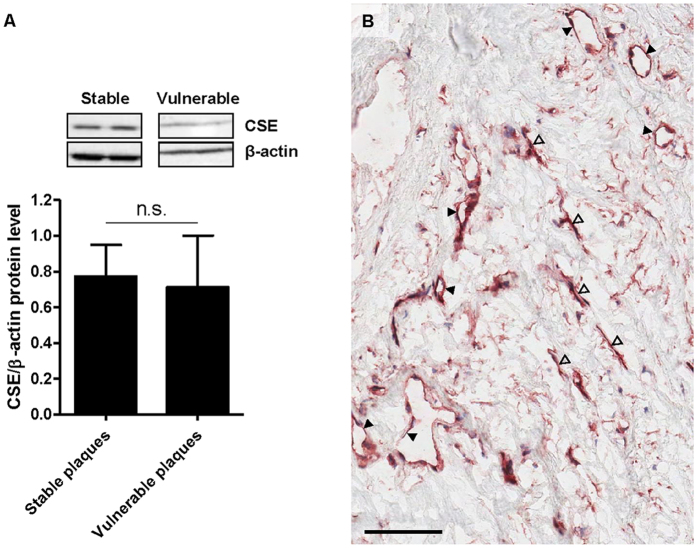
Carotid endarterectomy plaques express cystathionine γ-lyase (CSE). Representative western blot showing (variable) CSE protein expression and its quantitative analysis (n = 19 plaques) (**A**). No differences were observed between stable or vulnerable plaques. Photomicrograph of a CEA plaque showing positive immunohistochemical staining for CSE in microvascular structures (closed arrowheads), and elongated cells (open arrowheads) (**B**).

**Figure 3:**
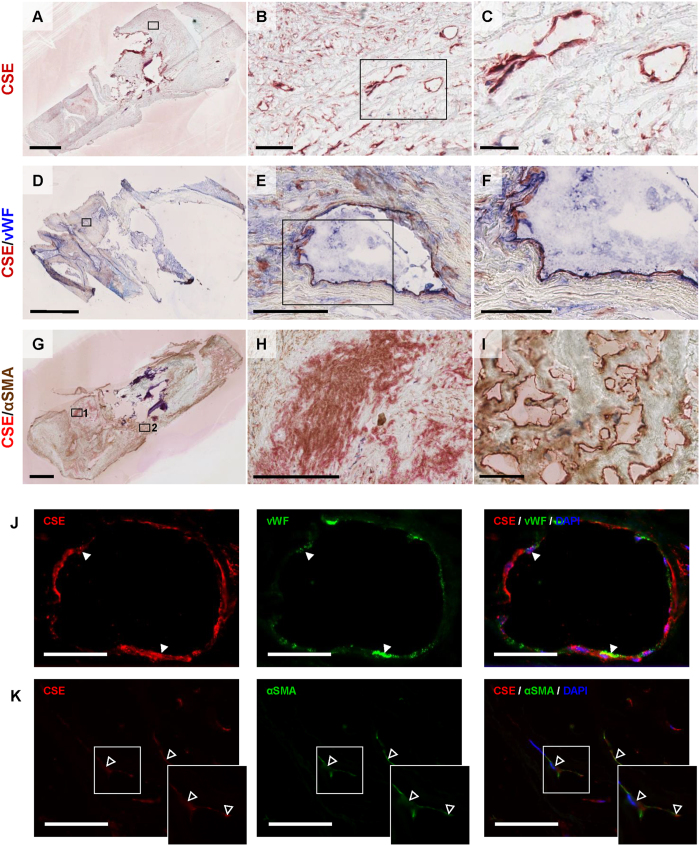
CSE is present in microvascular endothelial cells and αSMA^+^ cells. Representative photomicrographs of a plaque with CSE expression in intraplaque microvessels (**A**–**C**). Immunohistochemical double staining for CSE and vWF immunohistochemical staining, (**D**) showing double positivity in microvascular endothelial cells (**E**,**F**). CSE protein is visualized with AEC (red) and vWF with FastBlue (blue). Immunohistochemical double staining for CSE and αSMA (**G**), showing double positivity in intraplaque smooth muscle cells (**H**), but only CSE positivity in intraplaque microvessels (**I**). CSE protein is visualized with FastRed (red) and αSMA with DAB (brown). Scalebar: 2 mm (**A**,**D**,**G**); 100 μm (**B**,**E**,**H**); and 50 μm (**C**,**F**,**I**). Immunofluorescence double staining for CSE and vWF, showing co-localization in microvascular endothelial cells (closed arrowheads), 400× magnification, scalebar: 20 μm (**J**). Immunofluorescence double staining for CSE and αSMA, showing co-localization of CSE and αSMA in myofibroblast-like cells (open arrowheads), 400× magnification, scalebar: 20 μm (**K**).

In all plaques (n = 19), lumen narrowing, inflammatory cell infiltration, and microvessels were observed (Fig. 1). To determine intraplaque CSE protein expression levels, western blot analysis on both stable and vulnerable plaques was performed (Fig. 2A). No differences in CSE expression levels were observed between stable and vulnerable plaques. We also attempted to perform reproducible real time PCR analysis to determine intraplaque CSE mRNA expression levels. This was however not possible due to poor RNA quality with high A260/A280 ratio and low yields (data not shown), despite reproducible and reliable protein expression. To determine the localization of intraplaque CSE expression, immunohistochemistry was performed. As shown in Fig. 2B and Fig. 3A–C, CSE protein was localized within microvascular structures in CEA plaques. To confirm the endothelial phenotype of CSE-expressing cells, CSE/vWF immunohistochemical and immunofluorescence double labeling were performed. Double staining for CSE/vWF showed CSE expression in vWF^+^ endothelial cells (Fig. 3D–F,J) but also in cells of non-endothelial origin which appeared to be αSMA^+^ smooth muscle cells (Fig. 3G–K). Conjugate controls, as well as IgG_1_ isotype controls, were consistently negative (Suppl. Fig. 1).

### CSE siRNA knockdown in HMEC-1 significantly reduces CSE mRNA expression

**Figure 4:**
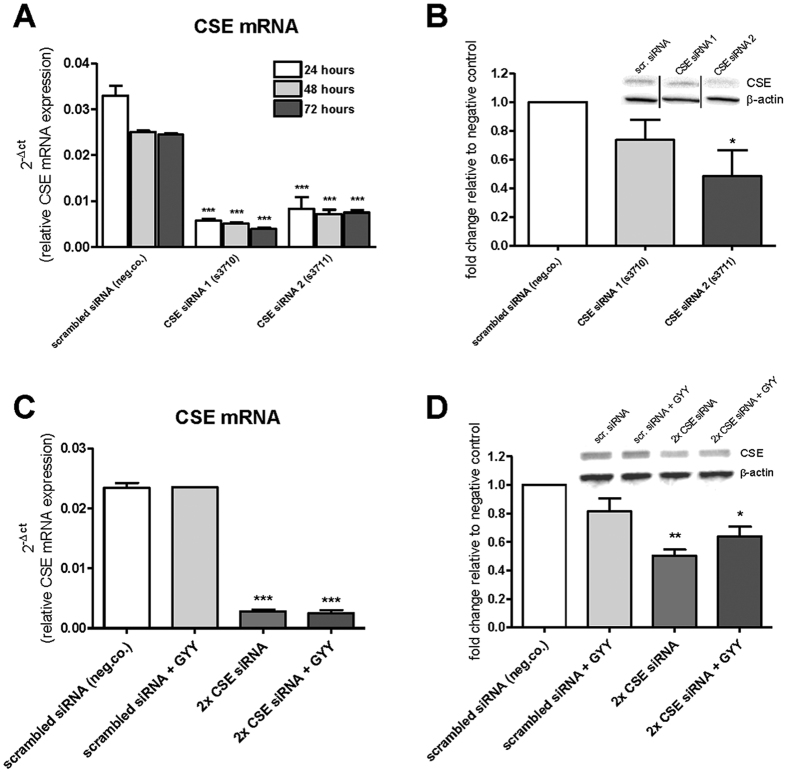
Confirmation of CSE knockdown with CSE siRNAs. HMEC-1 transfection with siRNA 1 (s3710) resulted in a dowregulation of CSE mRNA of 83%, 80% and 84%, and siRNA 2 (s3711) resulted in a dowregulation of 75%, 71% and 69% after 24, 48 and 72 hours respectively (**A**), n = 3, in which each replicate was performed in triplicate. Western blot analysis for CSE in HMEC-1 cells, showing a reduction of 26% (siRNA 1) and 51% (siRNA 2) of CSE protein expression (**B**) n = 3. qRT-PCR analysis for CSE in cultured HMEC-1 cells, 48 hours after transfection with two CSE siRNAs and addition of GYY4137, showing 88% (2× CSE siRNA) and 89% (2× CSE siRNA + GYY) knockdown of CSE mRNA expression compared to negative control. GYY4137 did not affect CSE mRNA expression (**C**), n = 3, in which each replicate was performed in triplicate. Western blot analysis for CSE in HMEC-1 cells, 48 hours after transfection with double CSE siRNA knockdown and addition of GYY4137, showing 45% (2× CSE siRNA) and 29% (2× CSE siRNA + GYY) knockdown of CSE protein expression compared to negative control (scrambled siRNA). GYY4137 did not affect CSE protein expression significantly (**D**) n = 3. Data are expressed as mean ± SEM of three independent siRNA transfection experiments. *P < 0.05, **P < 0.01, ***P < 0.00 vs. control. Data were analyzed using one-way ANOVA. Normality was tested using the Kolmogorov–Smirnov test. Bonferroni post-hoc analysis was applied to correct for multiple comparisons.

To study the effect of CSE on functional characteristics of HMEC-1 cells *in vitro* (*i.e.* tube formation, cell viability/proliferation, migration), CSE siRNA knockdown was established using two different CSE-specific siRNA’s. PCR analysis confirmed down-regulation of CSE mRNA in HMEC-1 cells 24, 48, and 72 hours after CSE mRNA knockdown. Both siRNA 1 (s3710) and siRNA 2 (s3711) resulted in a significant down-regulation of CSE mRNA expression (P < 0.001; Fig. 4A). Western blot analysis performed after 48 hours confirmed CSE knockdown, and revealed a decreased CSE protein expression with siRNA 1 (n.s.) and siRNA 2 (P < 0.05; Fig. 4B). In order to achieve more pronounced knockdown, double CSE knockdown was performed. To determine whether the potential effects of CSE knockdown resulted from reduced H_2_S production, exogenous H_2_S was added to the cultures by adding slow release H_2_S compound GYY4137. Double CSE knockdown resulted in 88% decreased CSE mRNA expression, which was unaffected by GYY4137 (Fig. 4C). At the protein level, double CSE knockdown resulted in 45% decreased CSE expression, which was not significantly affected by GYY4137 (Fig. 4D). CSE knockdown did not alter CBS or 3MST mRNA expression levels as evidenced by real-time PCR analysis (Suppl. Fig. 2).

### Inhibition of CSE decreases tube formation

**Figure 5:**
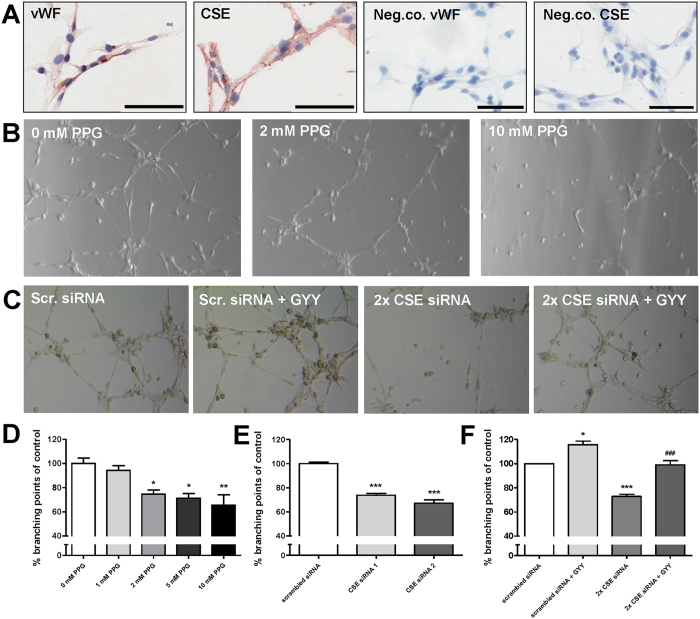
Inhibition of CSE in HMEC-1 cells reduces tube formation. Immunohistochemical staining of HMEC-1 cells for vWF and CSE. Conjugate controls were consistently negative. Scalebar: 100 μm (**A**) Representative photomicrographs showing tube formation assay with HMEC-1 cells in the presence of increasing concentrations (0–10 mM) of the irreversible CSE inhibitor PPG (**B**) transfected with scrambled siRNA (negative control), CSE siRNA 1 (s3710) and CSE siRNA 2 (s3711) and/or with slow release H_2_S donor GYY4137 (**C**) PPG dose-dependently decreases tube formation by 6%, 25%, 29%, and 34% with 1 mM, 2 mM, 5 mM, and 10 mM PPG respectively (**D**) CSE knockdown with siRNA 1 and siRNA 2 resulted in decreased tube formation of respectively 26% and 33% (**E**) GYY4137 significantly increased tube formation by 16%, double CSE knockdown inhibited tube formation with 27%, which was recovered by GYY4136 to 99% of control (**F**). For all tube formation experiments: n = 3, in which each replicate was performed in triplicate. Data are expressed as mean ± SEM. *P < 0.05, **P < 0.01, ***P < 0.001 *vs.* control ^###^P < 0.001 vs. 2× CSE siRNA. Data were analyzed using one-way ANOVA. Normality was tested using the Kolmogorov–Smirnov test. Bonferroni post-hoc analysis was applied to correct for multiple comparisons.

Cultured HMEC-1 cells were positive for vWF as well as CSE. Conjugate controls were consistently negative (Fig. 5A). HMEC-1 started to form tubes and capillary networks one hour after seeding, with maximal tube formation observed after 8 hrs. PPG was used to inhibit CSE activity. PPG dose-dependently inhibited tube formation up to 34% (Fig. 5B,D). PPG did not influence CSE mRNA expression (Suppl. Fig. 3). Single (one siRNA) and double (two siRNAs) CSE mRNA knockdown resulted in reduced tube formation (P < 0.001; Fig. 5C,E), which was restored to 99% of control after culture in the presence of slow release H_2_S donor GYY4137. GYY4137 without CSE knockdown stimulated tube formation with 16% (P < 0.05; Fig. 5F).

### Reduced CSE expression decreases cell viability

**Figure 6:**
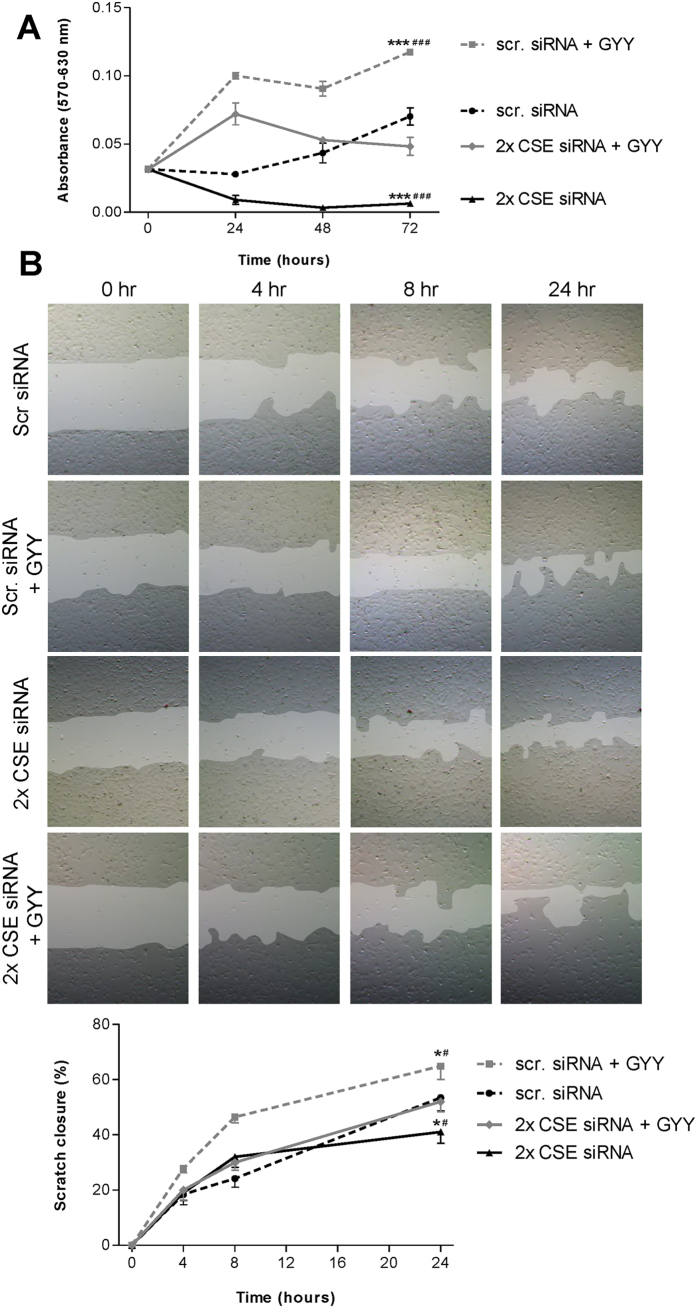
Inhibition of CSE in HMEC-1 cell viability and migration. In the MTT assay, double CSE mRNA knockdown resulted in inhibition cell viability of HMEC-1, compared to negative control (scrambled siRNA), and addition of 300 μmol/L GYY4137 was able to temporarily diminish this effect. Addition of GYY4137 alone resulted in induced mitochondrial activity compared to negative control (**A**). The MTT assay was repeated three times. For the scratch assay, representative pictures for each condition are shown to visualize scratch-closure over time. After 24 hours, 53% of the scratch was closed in the negative control conditions. Addition of GYY4137 significantly increased scratch closure at 24 hours, up to 65%. Double CSE knockdown decreased migration of HMEC, with a scratch closure of 41%, an effect which was reversed by addition of GYY with a scratch closure of 52% after 24 hours (**B**). Each condition in the scratch assay was repeated six times. Data are expressed as mean ± SEM. *P < 0.05, ***P < 0.001 vs. scr. siRNA; ^#^P < 0.05, ^###^P < 0.001 vs. 2× CSE siRNA + GYY. Data were analyzed using two-way ANOVA. Normality was tested using the Kolmogorov–Smirnov test. Bonferroni post-hoc analysis was applied to correct for multiple comparisons.

We next analyzed whether CSE knockdown also attenuated cellular processes related to angiogenesis, i.e. cell viability/proliferation and migration. Double (two siRNAs) CSE mRNA knockdown resulted in decreased cellular activity or viability compared to the scrambled siRNA control, as evidenced by lower absorbance in the MTT assay. Addition of GYY4137 significantly attenuated this effect. Administration of GYY4137 alone increased cell viability in HMEC-1 cells, compared to negative control (scrambled siRNA) (two-way-ANOVA, p < 0.001, Fig. 6A). These data indicate that CSE-derived endogenous H_2_S increases cell viability. The effect of CSE knockdown can be overcome by exogenous H_2_S.

### CSE knockdown inhibits HMEC-1 migration

Using a wound healing scratch assay double CSE mRNA knockdown decreased HMEC-1 migration when compared to the scrambled siRNA control. After 24 hours, 53% of the scratch was closed in the scrambled siRNA control, whereas after double CSE mRNA knockdown this was significantly lower (41%, two-way-ANOVA, p < 0.05). Addition of GYY4137 was able to diminish the effects of CSE knockdown (52% closure). GYY4137 alone resulted in increased cell migration (65% scratch closure) when compared with the scrambled siRNA control (two-way-ANOVA, p < 0.05, Fig. 6B). Similar to HMEC-1 tube formation and viability, these data indicate that CSE-derived endogenous H_2_S increases cell migration.

### CSE knockdown results in reduced expression of VEGFR2

**Figure 7:**
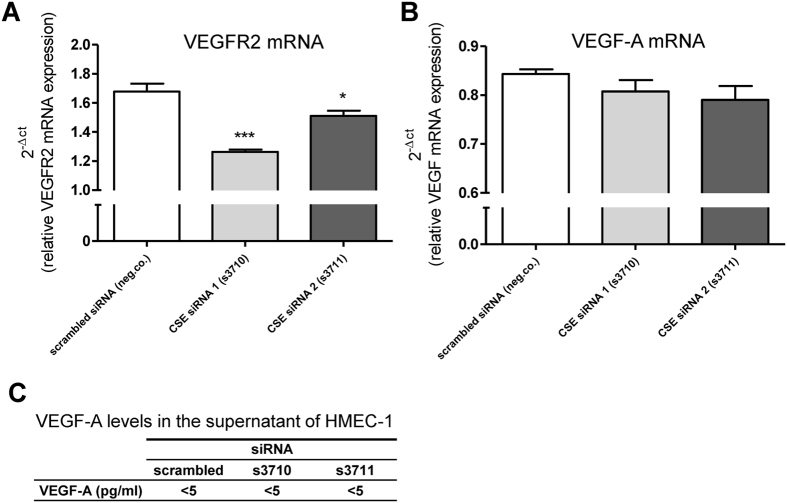
VEGFR2 and VEGF-A expression during CSE knockdown. qRT-PCR analysis for VEGFR2 in cultured HMEC-1 cells, showing 25% (CSE siRNA 1) and 10% (CSE siRNA 2) decreased VEGFR2 mRNA expression (**A**) n = 3, in which each replicate was performed in triplicate. qRT-PCR analysis revealed no differences in VEGF-A mRNA expression levels in HMEC-1 cells after transfection with different siRNAs (**B**) n = 3, in which each replicate was performed in triplicate. ELISA revealed undetectable low VEGF-A concentrations in supernatant of transfected cells, and showed no differences in VEGF-A concentration in HMEC-1 cells after transfection with different siRNAs (**C**), n = 3, in which each replicate was performed in duplicate. Data were analyzed using Kruskal Wallis test. Normality was tested using the Kolmogorov–Smirnov test. Dunns post-hoc analysis was applied to correct for multiple comparisons.

To study a possible pathway of pro-angiogenic effects of H_2_S, VEGF-A and VEGFR2 expression levels were analyzed in HMEC-1 cells. PCR analysis revealed significantly decreased VEGFR2 mRNA expression levels after CSE siRNA knockdown (Fig. 7A). No differences were observed for VEGF-A mRNA expression levels (Fig. 7B). Supernatant VEGF-A protein levels were below detection limit (Fig. 7C).

## Discussion

Major findings of this study are: 1) the detection of high levels of CSE in intraplaque microvessels in human CEA atherosclerotic plaques, 2) the stimulatory effect of CSE on angiogenesis of microvascular endothelium *in vitro*, and 3) CSE knockdown results in decreased expression levels of VEGFR2 mRNA after CSE knockdown. All together, these findings support a role for intraplaque H_2_S production as a risk factor for increased microvascular density, and thereby plaque vulnerability.

Unfortunately, we were not able to analyze intraplaque gene expression for CSE and the other H_2_S producing enzymes at the mRNA expression level. Due to large acellular areas of advanced plaques and degradation of RNA, the yield was very low and the mRNA quality rather poor. This is a technical issue, which has been described before^20^.

The tube formation assay with HMEC-1 cells was used as an *in vitro* model for micro-angiogenesis. Inhibition of CSE resulted in decreased tube formation, an effect that was rescued after adding GYY4137, a H_2_S donor. Similar results were obtained for HMEC-1 cell viability and migration. The observation that the functional phenotypes of CSE-deficient HMEC-1 cells can be rescued by exogenous H_2_S indicates that these CSE-dependent processes are H_2_S-mediated. The anti-angiogenic effects of CSE knockdown are possibly mediated via the VEGFR2 pathway. H_2_S can directly target VEGFR2^21^ but also indirectly as explained in more detail below. Our data indicate that human microvascular endothelial cell-mediated angiogenesis is, at least in part, regulated by H_2_S. Our results are in line with the previous reported pro-angiogenic actions of H_2_S^5^,^6^,^12^. However, here we describe pro-angiogenic effects of H_2_S on human microvascular endothelial cells *in vitro*. Intraplaque microvessels are composed of microvascular endothelial cells which prompted us to use human microvascular HMEC-1 cells; we believe this *in vitro* assay would be the best approximation of the *in vivo* situation. Since our *in vitro* data are in line with previous reports^5^,^6^,^12^, we assume that the underlying molecular mechanisms might also be similar. Although not determined by us in HMEC-1 cells, we propose that also in these cells H_2_S might promote angiogenesis involving pathways such as PI3K/AKT and MAPK pathways, as well as ATP-sensitive K-channels^6^. Recently, microRNA miR-640 was identified as a key regulator of the proangiogenic effects of H_2_S^22^. In response to H_2_S, expression of miR-640 was reduced in HUVECs and HMEC-1 cells through the VEGFR2-mTOR pathway resulting in increased HIF1A expression. HIF1A is a known regulator of angiogenic growth factors that promote angiogenesis eventually.

Composition of carotid atherosclerotic plaques, and its association with plaque rupture and cardiovascular events has been reported before^23^. Based on previous research, plaque neovascularization is associated with a more rupture-prone plaque phenotype and is increased in ruptured atherosclerotic plaques^24^. The predominant trigger for the development of plaque neovascularization are hypoxic conditions in the microenvironment of the necrotic core^25^. The intraplaque microvessels are presumed to originate from the adventitial vasa vasorum that infiltrate the damaged medial wall^26^. Here we clearly demonstrated CSE expression in intraplaque microvessels, which supports the concept that intraplaque produced H_2_S may further promote angiogenesis. Based on our data we cannot draw firm conclusions as to whether intraplaque hypoxia increases microvascular CSE expression. However, there is ample evidence for a role of increased demand of H_2_S in ischemic conditions. For example, inhibition of CSE in rats subjected to bilateral renal ischemia and reperfusion worsened renal function, indicating that the endogenous production of H_2_S is crucial in the recovery period after ischemia^27^. In mice subjected to ischemia and reperfusion, renal H_2_S production and plasma concentrations of H_2_S were increased after 24 hours, suggesting a reparative role for H_2_S after ischemia and reperfusion^28^. In line with this, we previously showed that in CSE knock out mice mortality is significantly higher when compared to wild type mice after subjection to bilateral ischemia and reperfusion^11^.

Also in more direct cell-based assays it was reported that hypoxia modulates CSE expression trough (post-)transcriptional regulation which may result in increased CSE protein levels^29^. We therefore propose that intraplaque hypoxia is likely to be responsible for preserved or even increased CSE expression in intraplaque microvessels.

Inhibiting microvessel formation in atherosclerotic plaques may be a strategy to reduce the risk for plaque rupture. Since angiogenesis is induced by CSE-derived H_2_S, this pathway seems a good candidate for targeted therapeutic inhibition. However, the therapeutic potential of systemic CSE inhibition is hampered by the fact that CSE and H_2_S have systemic anti-atherogenic effects, including the scavenging of reactive oxygen species, inhibition of vascular smooth muscle cell proliferation, and reduction of inflammation^15^,^30^. Overexpression of CSE^31^, exogenous administration of NaHS (a fast H_2_S donor),^14^ or GYY4137 (a slow release H_2_S donors)^32^ showed reduction of atherosclerotic plaque size in ApoE^−/−^ mice. In line with this, atherosclerosis is accelerated in CSE deficient mice^16^. CSE deficiency is also accompanied by a lower vessel density in an ischaemic limb model in mice, an effect which was reduced with H_2_S donor diallyl trisulfide^33^. Based on these published data and our data presented here, local intraplaque CSE inhibition with preserved systemic CSE activity is required. It has been shown that CSE and VEGF both act via a common pathway in angiogenesis as VEGF-stimulated growth of microvessels in mouse aortic rings is dependent on the presence of active CSE^12^. In addition, it has been shown that H_2_S could directly activate VEGFR2^21^ as already eluded to above. Local treatment with soluble vascular endothelial growth factor receptor-1 (sFlt-1) as a VEGF inhibitor was shown to be a promising treatment modality against plaque development and progression in rabbits^34^. However, systemic VEGF inhibition using the selective VEGFR2 blocker PTK787 in ApoE^−/−^ mice augmented atherosclerotic plaque development compared to placebo treatment^35^. These data underscore the importance of local *vs.* systemic inhibition of angiogenesis and should be taken into account when considering CSE as a potential target for intervention. As miR-640 is identified as a key regulator of the proangiogenic effects of H_2_S^22^ also locally (intraplaque) increased expression of miR-640 (or miRs with similar effects) might be considered to reduce H_2_S-mediated angiogenesis.

Our hypothesis contrasts earlier described atheroprotective effects of the CSE/H_2_S pathway in atherogenesis. Based on our data we propose that the CSE/H_2_S pathway might be protective in the early phase of plaque formation by e.g. attenuating endothelial dysfunction and reducing inflammation. However, during plaque progression CSE/H_2_S might actually promote plaque vulnerability due to its pro-angiogenic effects. One should be aware of this potential risk when considering H_2_S as treatment modality for atherosclerosis.

In conclusion, we here demonstrate microvessel-associated CSE expression in human atherosclerosis. Although microvessel formation might be beneficial for cells to survive hypoxia in the expanding plaque, it may actually increase plaque vulnerability. High levels of CSE in atherosclerotic plaques may be a potential risk for plaque vulnerability through the local production of H_2_S.

## Methods

### Carotid endarterectomy specimens

Atherosclerotic plaques were obtained from patients undergoing carotid endarterectomy (CEA) for atherosclerotic carotid disease. Patients were referred to as symptomatic when suffering from stroke, transient ischemic attack or amaurosis fugax. CEA was performed when internal carotid artery stenosis was >70%. CEA specimens were divided into different parts that were either snap frozen at −80 °C or fixed in formaldehyde and paraffin-embedded. The study was performed conform to the principles of the Declaration of Helsinki and approved by the Institutional Review Board. All patients provided informed consent.

### Immunohistochemistry and immunofluorescence

Histochemical stainings were performed on paraffin sections of all plaques to assess general morphology (PAS staining), intraplaque hemorrhage and fibrous cap thickness (Martius, Scarlet and Blue; MSB staining).

For immunostaining, frozen sections were fixed in 100% acetone, and cultured HMEC-1 cells were fixed in 90% acetone/10% dH_2_O at −20 °C for 10 minutes. Sections or cells were subsequently stained for human CSE (mouse IgG_1_ κ monoclonal, Abnova, clone S51), human vWF (rabbit polyclonal, Dako, ref: A0082), human αSMA (mouse IgG_2a_ κ monoclonal, Dako, ref: M0851), human CD34 (mouse IgG_1_ κ monoclonal, Dako, clone QBEnd-10, code number M7165), or human CD68 (mouse IgG_3_ κ monoclonal, Dako, clone KP-1). Following incubation with primary antibodies (1 hr, room temperature), sections/cells were incubated with HRP-conjugated rabbit-anti-mouse IgG (CSE and αSMA) or goat-anti-rabbit IgG (vWF) polyclonal antibodies (Dako). 3-amino-9-ethylcarbazole (AEC) or 3,3′-diaminobenzidine (DAB) was used as chromogen, and nuclei were counterstained using hematoxylin.

For CSE/vWF immunohistochemical double staining, frozen sections of plaques were fixed in 100% acetone, and incubated with mouse IgG_1_ anti-human-CSE for 1 hour at room temperature. Sections were subsequently incubated with goat-anti-mouse IgG_1_ biotin labeled, and streptavidin-HRP. HRP-activity was visualized using AEC. Sections were then incubated with the rabbit anti-human-vWF for another hour, followed by incubation of goat-anti-rabbit-alkaline phosphatase and stained using FastBlue (Life Technologies, Bleiswijk, The Netherlands). For αSMA/CSE immunohistochemical double staining, frozen sections of plaques were fixed in 100% acetone, and incubated with mouse IgG_2a_ anti-human-αSMA for 1 hour, followed by goat-anti-mouse IgG_2a_-HRP. HRP-activity was visualized using DAB. After that, sections were incubated with the second primary antibody, mouse IgG_1_ anti-human-CSE for 1 hour, followed by incubated with goat-anti-mouse IgG_1_ biotin labeled, and streptavidin-alkaline phosphatase and stained using FastRed. Nuclei were counterstained using hematoxylin.

Immunofluorescent double labeling for CSE and vWF or CSE and αSMA was performed as described above using goat-anti-mouse-IgG1-TRITC (for CSE) and goat-anti-rabbit-FITC (for vWF) or goat-anti-mouse-IgG2a-FITC (for αSMA) (SouthernBiotech) as secondary antibodies. Nuclei were counterstained with DAPI. Primary antibody was replaced by PBS in negative controls.

### Plaque vulnerability score

All 19 carotid atherosclerotic plaque specimens were assessed for vulnerability. For plaque vulnerability score, the following criteria were taken into account: microvessel density, thickness of the fibrous cap, CD68^+^ macrophage infiltration and the presence of intraplaque hemorrhage. Intraplaque microvessel density was determined in two hotspots. A hotspot is defined as one field of view at 200 × magnification. CD34^+^ microvessels were manually counted in two hotspots, and mean number of CD34^+^ microvessels per mm^2^ was calculated. Microvessel density resulted in the following vulnerability score; (0) less then 10 microvessel per mm^2^, (1) between 10 and 20 microvessels per mm^2^, and (2) more then 20 microvessels per mm^2^. Fibrous cap thickness was based upon the MSB staining. The visually thinnest part of the fibrous cap was measured, and scored according to the Critical Cap Thickness criteria;^19^ (0) the thinnest part of the fibrous cap was >200 μm, and (1) the thinnest part of the fibrous cap was <200 μm. Inflammation in the plaque was assessed by measurement of CD68^+^ macrophages. CD68 stained sections were scanned using the NanoZoomer 2.0HT Digital slide scanner (Hamamatsu, Japan) and analyzed for positive pixel area (CD68) using the Aperio Positive Pixel Analysis v9.1 algorithm. CD68 staining resulted in the following vulnerability score; (0) less then 2% CD68 positive pixel area, and (1) more then 2% CD68 positive pixel area. Finally, intraplaque hemorrhage was determined, based on the MSB staining and resulted in the following vulnerability score; (0) no detectable intraplaque hemorrhage, and (2) detectable intraplaque hemorrhage. The total sum of the scores was calculated to a total vulnerability score. A score lower then 4 was defined as stable plaque, and a score of 4 or higher was defined as vulnerable plaque.

### HMEC-1 cell culture

Human dermal microvascular endothelial cells (HMEC-1) were kindly provided by Dr. E.W. Ades (CDC, Atlanta, USA)^36^ via Prof. G. Molema and the UMCG Endothelial Cell Facility. Cells were cultured in M-199 medium (Lonza) supplemented with 10% (v/v) FCS (HyClone), 10% (v/v) human serum (Lonza), 2 mM L-glutamine (Lonza) and 100 U/ml penicillin and 100 μg/ml streptomycin (Lonza). Cells were expanded in T75 flasks (Corning) at 37 °C and 5% CO_2_ in a humidified atmosphere. For immunophenotyping, cells were cultured on coverslips. Tube formation assays were performed as described below.

### CSE knockdown in HMEC-1

Endogenous H_2_S production by CSE was downregulated by transfection with small interfering RNA (siRNA). Two different CSE siRNAs were used for this purpose (Silencer^®^ Select siRNA, s3710 and s3711, Life Technologies). Scrambled siRNA was used as a negative control (Silencer^®^ Select Negative Control No. 1 siRNA 4390843). Experiments were performed following the user’s guide. Briefly, 200 μl opti-MEM reduced medium (Gibco^®^, Life Technologies) per well was added in a 12-well plate. To each well 4.8 μl siRNA (2.5 μmol/L) was added, as well as 2 μl Lipofectamine RNAiMAX (Life Technologies). Next, 1 ml of cell-suspension was added per well, resulting in a final concentration of 10 nmol/L siRNA. After 24, 48, and 72 hours, cells were harvested for PCR analysis and western blot analysis. In order to accomplish more pronounced knockdown, double CSE knockdown with the 2 CSE siRNAs was performed. For double CSE knockdown experiments 200 μl opti-MEM reduced medium per well was added in a 12-well plate. To each well either 4.8 μl scrambled siRNA (2.5 μmol/L) and 2 μl Lipofectamine RNAiMAX was added per well, or 4.8 μl CSE siRNA 1 and 4.8 μl CSE siRNA 2 and 4 μl Lipofectamine RNAiMAX was added per well. Next, 1 ml of cell-suspension was added per well, resulting in a final concentration of 10 nmol/L per siRNA. 12 hours after transfection, GYY4137 (30 mM) was administered to a final concentration of 300 μM GYY4137.

### HMEC-1 tube formation

A Matrigel tube formation assay was performed using HMEC-1 cells to study angiogenic capacity of microvascular endothelial cells. Ten μl growth factor-reduced Matrigel (BD Biosciences) was allowed to polymerize at 37 °C for 30 minutes in Angiogenesis μ-slides (Ibidi GmbH). HMEC-1 cells were plated on the Matrigel surface (5000 cells/well in 50 μl serum-free medium). To inhibit CSE activity, DL-propargylglycine (PPG, Sigma-Aldrich) was used. Tube formation was performed in presence of CSE inhibitor PPG using various concentrations (0, 1, 2, 5 and 10 mmol/L) in order to inhibit CSE activity. To inhibit CSE mRNA expression two different siRNAs (single or in combination) were used as described above and tube formation was analyzed 40 hours after transfection. Slow release H_2_S compound GYY4137 (kindly provided by M. Whitemann, Exeter, UK) was used as an H_2_S donor in a concentration of 300 μmol/L^37^. After 8 hours, images were taken from 5 selected areas per well in a standardized way. The total number of branching points in the 5 images was determined using Aperio Imagescope software (Aperio Technologies). The tube formation assay was repeated three times, in which each replicate was performed in triplicate.

### MTT cell viability assay

To examine cell viability and mitochondrial activity of HMEC-1, a 3-(4,5-dimethylthiazol-2-yl)-2,5-diphenyl tetrasodium bromide (MTT) assay was used according to the manufacturer’s instruction. In short, HMEC-1 were transfected with siRNA and seeded into 96-well plates (2,500 cells per well). MTT reagent (10 μl) was added after one hour (t = 0), after 24 hours (t = 1), 48 hours (t = 2) or 72 hours (t = 3). Four hours after MTT administration, formazan crystals were formed, 100 μl isopropanol with 0.04 N HCl was added and thoroughly mixed to solubilize formazan. Directly after solubilization, the absorbance was measured at 570 nm and a reference wavelength of 630 nm (Varioskan, Thermo Scientific). The MTT assay was repeated three times.

### HMEC-1 scratch assay

Migration activity of HMEC-1 cells was studied using the scratch assay. Cells were seeded in 24-well plates to confluence. 24 hours after transfection with siRNAs, a scratch was made with a pipette tip of a 200 μl pipette. The medium was directly changed into serum-free medium and photomicrographs were taken (t = 0). Pictures were taken at 4 hours (t = 1), 8 hours (t = 2), and 24 hours (t = 3). Migration potential was expressed as percentage of scratch closure. Each condition in the scratch assay was repeated six times.

### Western blot analysis

Protein was isolated from CEA cryosections (30 × 10 μm) as well as from cultured HMEC-1 cells using RIPA Buffer supplemented with 1% Halt Phosphatase inhibitor cocktail and 1% Halt Protease inhibitor cocktail, 0.1 mmol/L phenylmethylsulfonyl fluoride (PMSF) and 20 μg/ml trypsin inhibitor. Total protein content of the lysates was determined with the pyrogallol red method as previously described^38^. Briefly, 5 μl of sample was added in a 96-well plate (Corning), 300 μl pyrogallol red-molybdate complex was then added. After 10 minutes absorbance was measured at 600 nm (Varioskan, Thermo Scientific) and protein concentration was calculated against a calibration curve. Equal amounts of protein were boiled and electrophoretically separated in 10% SDS-PAGE gel and transferred to a nitrocellulose membrane. Aspecific binding of antibodies was blocked using 5% non-fat milk powder in Tris-buffered saline-0.1% Tween-20 (TBST) for 1 hour. Membranes were incubated with primary antibody overnight at 4 °C (for CSE; 1:500, mouse monoclonal IgG_1κ_, Abnova). β-actin (1:1000, mouse monoclonal IgG_1_, Santa Cruz Biotechnology) served as housekeeping protein. Horseradish peroxidase-labeled antibody was used as a secondary antibody. All antibody incubations were followed by short washing with TBST. Immunoreactions were visualized by ECL Western Blotting Substrate (Thermo scientific, Waltham, MA, USA) and images were taken with the Bio-Rad-ChemiDoc MP system and quantified using ImageJ software.

### Real-time PCR analysis

RNA was isolated from cultured HMEC-1 cells using TRIzol Reagent (Invitrogen). 1 ug RNA was converted to cDNA using SuperScript II reverse transcriptase and random hexamere primers (Life Technologies). CSE (assay Hs00542284_m1), CBS (assay Hs00163925_m1), 3MST (assay Hs00560401_m1), VEGF-A (assay Hs00900055_m1) and VEGFR2 (assay Hs00911700_m1) mRNA expression was measured with a Taqman Gene expression assay (Applied Biosystems). For normalization, TATA box binding protein (TBP) was included as housekeeping gene using the following primers (FW: GCCCGAAACGCCGAATAT; REV: CCGTGGTTCGTGGCTCTCT) and probe (ATCCCAAGCGGTTTGCTGCGG) (Eurogentec). Reactions were performed on an ABI7900HT thermal cycler (Applied Biosystems). The comparative Ct method (2^−ΔCt^ method) was used to calculate relative gene expression.

### ELISA

Human VEGF-A was measured in supernatant of cultured HMEC-1 48 hours after transfection with different siRNAs (Quantikine ELISA Kit, Catalog# DVE00; R&D Systems). Analysis was performed according to the user’s guide. In short; 50 μl Assay Diluent and the standards, blanks and samples (200 μl) were added to each well. After 2 hours incubation, wells were washed three times followed by addition of 200 μl Conjugate and incubation for 2 hours. After 3 times washing, 200 μl of Substrate Solution was added and incubated at room temperature in the dark. After 20 minutes, 50 μl Stop Solution was added per well and within 30 minutes absorbance was measured at 450 nm (Varioskan, Thermo Scientific). VEGF-A concentration was then calculated by using the calibration curve.

### Statistical analysis

All data were analyzed with GraphPad PRISM 5.0 (GraphPad, San Diego, CA, USA). Data were analyzed using one-way ANOVA or Kruskal Wallis test where appropriate. Normality was tested using the Kolmogorov–Smirnov test. Bonferroni or Dunns post-hoc analysis was applied to correct for multiple comparisons. Data are shown as mean ± SEM (Standard Error of the Mean). Differences were considered statistically significant when *P* < 0.05.

## Additional Information

**How to cite this article**: van den Born, J. C. *et al*. Cystathionine γ-lyase is expressed in human atherosclerotic plaque microvessels and is involved in micro-angiogenesis. *Sci. Rep.* **6**, 34608; doi: 10.1038/srep34608 (2016).

## Supplementary Material

Supplementary Information

## Figures and Tables

**Table 1:** Patient characteristics.

	Overall n = 19	Stable n = 11	Vulnerable n = 8	P-trend
Demographics				
Age (years)	72.2 ± 8.1	73.4 ± 8.5	71.2 ± 7.3	0.56
Male gender	15 (79%)	9 (82%)	6 (75%)	0.72
BMI (kg/m^2^)	28.7 ± 3.5	29.5 ± 3.0	27.6 ± 4.0	0.25
Smoking*	8 (42%)	3 (27%)	5 (63%)	0.26
Symptomatology	15 (79%)	8 (73%)	7 (88%)	0.44
Symptoms - surgery interval (days)†	25 ± 14	23 ± 10	27 ± 18	0.64
Clinical parameters				
SBP (mmHg)	146 ± 14	145 ± 15	147 ± 12	0.86
DBP (mmHg)	72 ± 12	75 ± 12	68 ± 10	0.18
Serum creatinine (μmol/L)	91 ± 17	88 ± 13	96 ± 22	0.36
Co-morbidity				
Diabetes mellitus	5 (26%)	2 (18%)	3 (38%)	0.25
Hypertension	18 (95%)	11 (100%)	7 (88%)	0.39
Hyperlipidemia	16 (84%)	10 (91%)	6 (75%)	0.35

Data are presented as mean ± SD or n (%).

^*^Smoking is presented as current smokers including abstinence <1 year.

^†^Symptoms - surgery interval is presented as days from first symptoms until day of surgery (only in symptomatic patients).
