# Supplementary material for: Cystathionine γ-lyase is expressed in human atherosclerotic plaque microvessels and is involved in micro-angiogenesis
Source: Sci Rep. 2016 Oct 6;6:34608. doi: 10.1038/srep34608 (PMC5052587; doi:10.1038/srep34608)
Supplement: Supplementary Information [file srep34608-s1.pdf]

Cystathionine γ-lyase is expressed in human atherosclerotic plaque microvessels and is involved in micro-angiogenesis

J.C. van den Born, MD; R. Mencke, BSc; S. Conroy, MSc; C.J. Zeebregts, MD PhD; H. van Goor, PhD; and J.L. Hillebrands, PhD

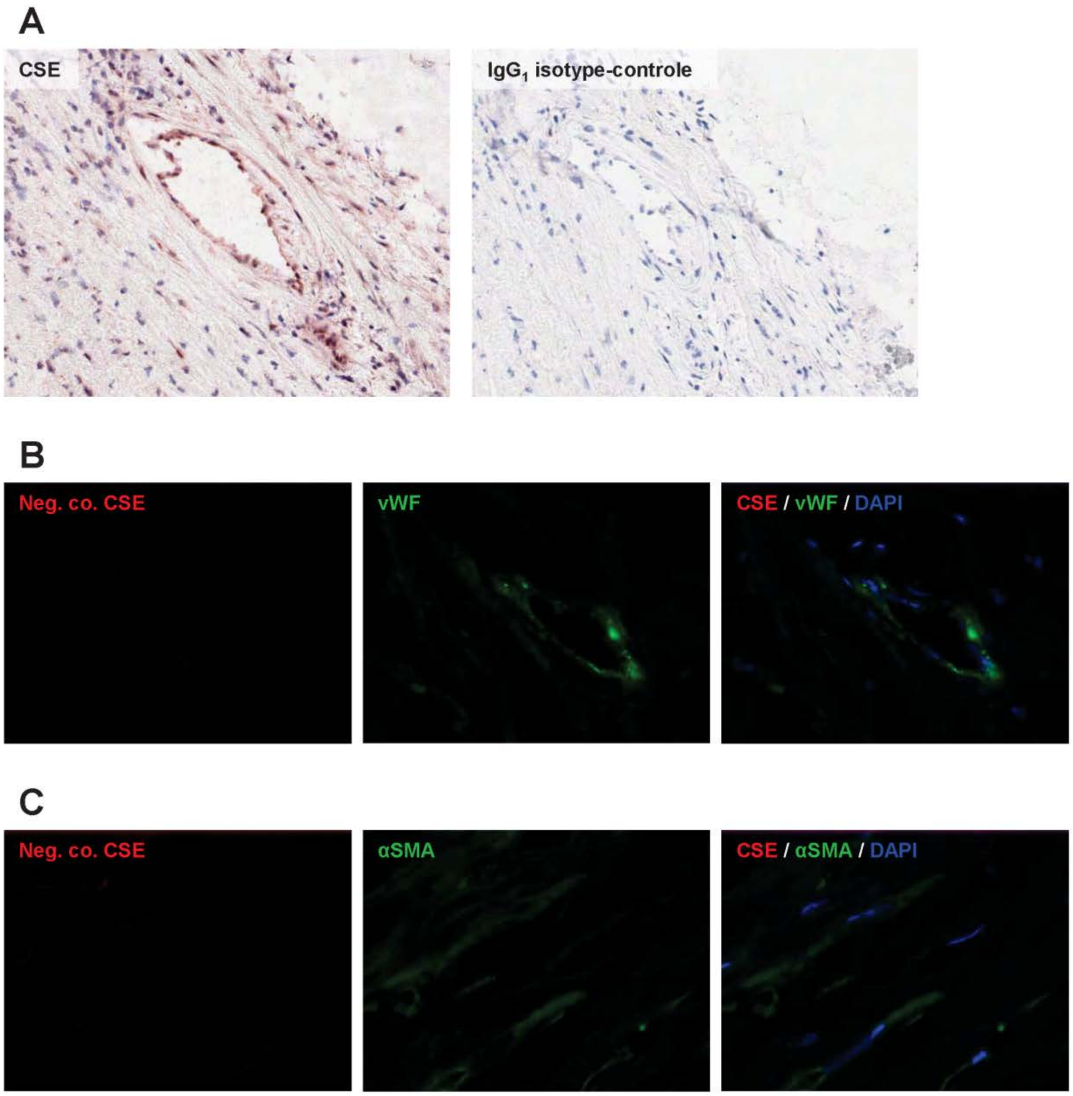

Supplemental figure 1: Negative control immunohistochemical and immunofluorescence stainings.

Positive signal (intraplaque microvessel) and negative control of carotid endarterectomy plaque for CSE staining. First antibody was replaced by isotype control (mouse IgG_1 monoclonal), normal staining protocol was further applied (A). Negative control staining of CSE/vWF double immunofluorescence staining (B) and CSE/αSMA double immunofluorescence staining (C) was performed, only vWF or αSMA antibody was used, and CSE antibody was replaced by PBS supplemented with 1% bovine serum albumin. Staining protocol was applied as decribed. 400x magnification.

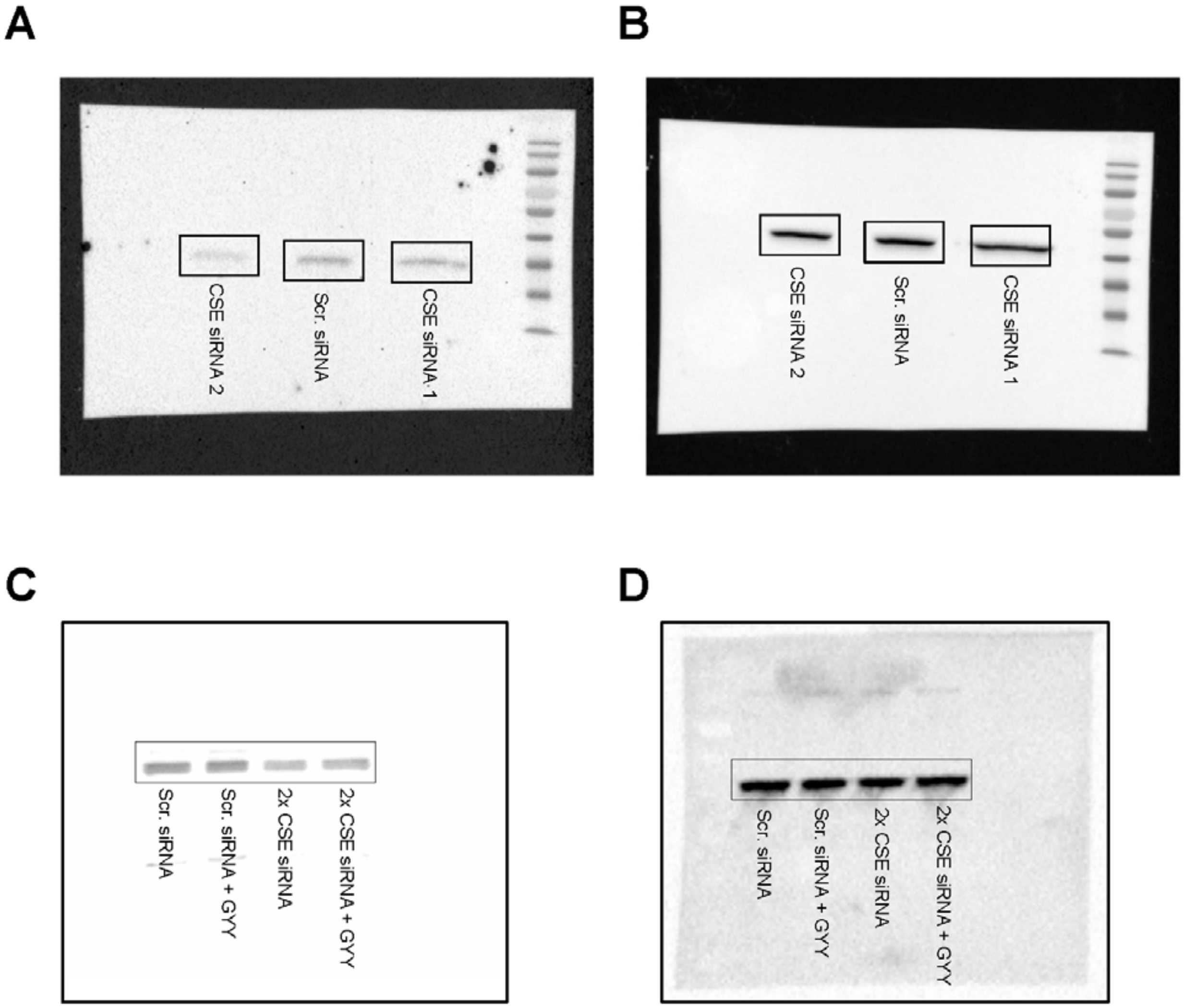

Supplemental figure 2: Original scans of western blots.
Unprocessed original scans of western blots of siRNA-treated HMEC-1 for CSE (A) and ß-actin (B). Original scans of western blots of HMEC-1 cells after siRNA silencing and/or GYY4137 treatment, for CSE (C) and ß-actin (D). Marked bands were used for figure 4.

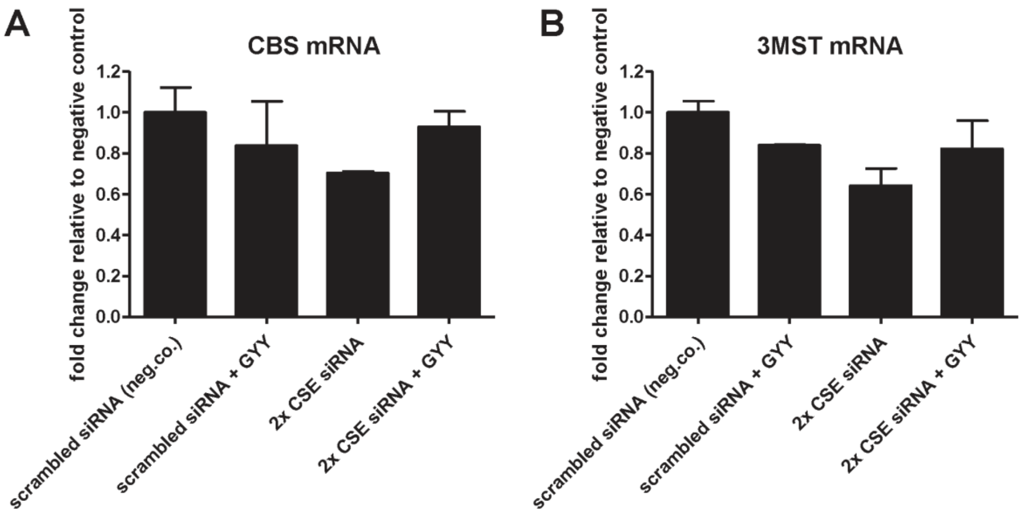

Supplemental figure 3: No differences in CBS and 3MST mRNA expression with siRNA CSE silencing.
Real-time PCR analysis revealed no differences in CBS (A) and 3MST (B) mRNA expression levels in HMEC-1 cells after siRNA silencing of CSE and addition of slow release H_2S compound GYY4137. n=3, in which each replicate was performed in triplicate.

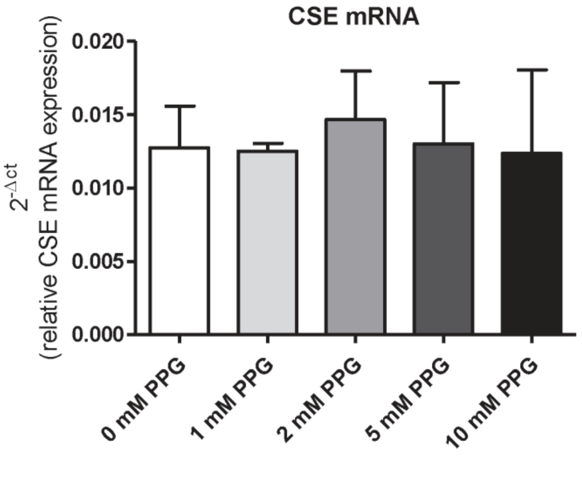

Supplemental figure 4: No differences in CSE mRNA expression during inhibition of CSE activity with PPG.
Real-time PCR analysis revealed no differences in CSE mRNA expression levels in HMEC-1 cells with different concentrations of PPG treatment. n=3, in which each replicate was performed in triplicate.
